# Heterogeneous treatment effects of adjuvant therapy for patients with cervical cancer in the intermediate‐risk group

**DOI:** 10.1002/cam4.6460

**Published:** 2023-08-16

**Authors:** Ayumi Taguchi, Kosuke Kato, Konan Hara, Akiko Furusawa, Yujiro Nakajima, Chihiro Ishizawa, Michihiro Tanikawa, Kenbun Sone, Mayuyo Mori, Muneaki Shimada, Aikou Okamoto, Munetaka Takekuma

**Affiliations:** ^1^ Department of Obstetrics and Gynecology, Graduate School of Medicine The University of Tokyo Tokyo Japan; ^2^ Gynecology, Tokyo Metropolitan Cancer and Infectious Diseases Center Komagome Hospital Tokyo Japan; ^3^ Department of Economics University of Arizona Tucson Arizona USA; ^4^ Department of Gynecology Shizuoka Cancer Center Hospital Shizuoka Japan; ^5^ Department of Radiological Sciences Komazawa University Tokyo Japan; ^6^ Department of Obstetrics and Gynecology Tohoku University School of Medicine Sendai Japan; ^7^ Department of Obstetrics and Gynecology The Jikei University School of Medicine Tokyo Japan

**Keywords:** adjuvant chemotherapy, adjuvant radiotherapy, cervical cancer, cohort studies, propensity score, treatment outcome

## Abstract

**Background:**

The efficacy of adjuvant therapy for patients with cervical cancer with intermediate risk (CC‐IR) remains controversial. We examined the impact of adjuvant therapy on survival outcomes in patients with CC‐IR and evaluated the heterogeneous treatment effects (HTEs) of adjuvant therapies based on clinicopathologic characteristics.

**Methods:**

We retrospectively analyzed a previous Japanese nationwide cohort of 6192 patients with stage IB–IIB cervical cancer who underwent radical hysterectomy. We created two pairs of propensity score‐matched treatment/control groups to investigate the treatment effects of adjuvant therapies: (1) adjuvant therapy versus non‐adjuvant therapy; (2) chemotherapy versus radiotherapy conditional on adjuvant therapy. Multivariate analyses with treatment interactions were performed to evaluate the HTEs.

**Results:**

Among the 1613 patients with CC‐IR, 619 and 994 were in the non‐treatment and treatment groups, respectively. Survival outcomes did not differ between the two groups: 3‐year progression‐free survival (PFS) rates were 88.1% and 90.3% in the non‐treatment and treatment groups, respectively (*p* = 0.199). Of the patients in the treatment group, 654 and 340 received radiotherapy and chemotherapy, respectively. Patients who received chemotherapy had better PFS than those who received radiotherapy (3‐year PFS, 90.9% vs. 82.9%, *p* = 0.010). Tumor size was a significant factor that affected the treatment effects of chemotherapy; patients with large tumors gained better therapeutic effects from chemotherapy than those with small tumors.

**Conclusion:**

Adjuvant therapy is optional for some patients with CC‐IR; however, chemotherapy can be recommended as adjuvant therapy, particularly for patients with large tumors.

## 1 INTRODUCTION

Cervical cancer is a common gynecologic malignancy worldwide. In Japan, 10,879 women were newly diagnosed with cervical cancer in 2019, and 2887 patients died of the disease in 2020.^1^, ^2^ Standard therapeutics for International Federation of Gynaecology and Obstetrics (FIGO) 2018 stage IB–IIA cervical cancer are radical hysterectomy and definitive radiation‐based therapies.^3^ Based on the postoperative pathological diagnosis, patients are categorized into three groups according to recurrence risk: high‐risk with lymph node metastasis (LNM) or parametrium invasion; intermediate‐risk with deep interstitial invasion, large size, or lymphovascular space invasion (LVSI); and low‐risk without any recurrence risk.^4^ For patients in the high‐risk group, adjuvant chemoradiotherapy (CRT) is recommended to prevent recurrence.^5^, ^6^ However, adjuvant radiation‐based therapies sometimes cause severe life‐threatening toxicities compared to surgery alone.^6^, ^7^ Therefore, adjuvant chemotherapies have been attracting attention as alternative strategies to radiation‐based adjuvant therapies.^8^, ^9^ Currently, a randomized Phase III trial comparing adjuvant chemotherapy and CRT in high‐risk group patients is being conducted in Japan.^8^ However, not only types of adjuvant therapy but the indications of adjuvant therapies for patients with cervical cancer with intermediate‐risk (CC‐IR) group remain controversial.^7^, ^9^, ^10^, ^11^ Based on a prospective randomized control trial from the Gynecologic Oncology Group, adjuvant radiotherapy significantly improved progression‐free survival (PFS), whereas a significant impact on overall survival (OS) was not confirmed.^7^ Particularly, the prognoses of patients with CC‐IR worsened as the number of risk factors increased.^12^, ^13^ Moreover, a previous study demonstrated that pelvic radiotherapy improved the survival outcomes of patients with CC‐IR with two or more risk factors.^14^ According to Japanese clinical practice guidelines for cervical cancer, adjuvant therapies are proposed based on recurrence risk factors^15^; however, the indications and types of adjuvant therapies for CC‐IRs differ among institutions.^16^, ^17^

Due to the wide variety of patient and tumor characteristics observed in CC‐IR, the effectiveness of adjuvant therapy might vary among patients. Therefore, we focused on the heterogeneous treatment effects (HTEs) of adjuvant therapies in patients with CC‐IR. HTE analysis focuses on investigating different treatment effects in individuals or subgroups in a population.^18^, ^19^, ^20^ Therefore, by conducting a HTE analysis, it may become possible to identify CC‐IR subgroups that benefit from adjuvant therapies. This study aimed to examine the impact of adjuvant therapies on the survival outcomes of patients with CC‐IR and identify groups that particularly benefit from adjuvant therapies using an HTE analysis. Simultaneously, we also focused on the HTEs of chemotherapy compared with radiation‐based therapies.

## 2 METHODS

### 2.1 Eligibility

This study was a secondary analysis of the Japanese Gynecologic Oncology Group (JGOG) dataset (JGOG1072S) generated by a previous study investigating the prognosis of cervical cancer patients who received radical hysterectomy.^13^, ^21^ The JGOG1072S study was a nationwide, large‐scale retrospective observational study conducted in 116 JGOG‐designated institutions. The survey collected consecutive cases of FIGO 2008 stage IB–IIB cervical cancer treated with primary radical hysterectomy between January 1, 2004, and December 31, 2008. The survey period for data collection was from October 1, 2012, to February 28, 2013. Institutional review board approval was obtained from Tottori University, which served as the host institution, and the JGOG‐participating institutions reviewed the protocol.

From the JGOG1072S database, we extracted patients with CC‐IR based on the Japanese Society of Gynecologic Oncology guidelines as follows.^15^ First, we excluded patients with distant metastasis, those who received neoadjuvant chemotherapy, and those with incomplete data. CC‐IR was defined as follows: cervical cancer without pathological LNM or parametrium infiltration and with one or more recurrent risk factors, including positive LVSI, large size (≥4 cm), and deep stromal invasion to the outer half (excluding low‐risk cases). Institutional review board approval for this study was obtained from the University of Tokyo (approval number: 2021078NI‐2) and Tokyo Metropolitan Komagome Hospital (approval number: 2749). The institutional review board granted an opt‐out recruitment approach and waived the need for obtaining written informed consent from each patient. We adhered to the Declaration of Helsinki.

### 2.2 Variables

The data collected included age, institution, pathology, tumor size, the presence or absence of LNM, parametrial tumor involvement, stromal invasion to the outer half, LVSI, uterine corpus invasion, vaginal invasion, PFS and OS rates, and the context of adjuvant therapy. Age was categorized as <40, 40 to <50, 50 to <60, 60 to <70, and ≥70 years; tumor size was categorized as <2 cm, 2 to <4 cm, and ≥4 cm. The pathological information consisted of histological evaluation (squamous cell carcinoma [SCC] or non‐SCC). Institutions were categorized into two groups: high‐volume and non‐high‐volume centers. High‐volume centers were defined as the top 10 institutions in terms of registered cases since the number of enrollments at these institutions accounted for one fourth of the total enrollment.

**FIGURE 1:**
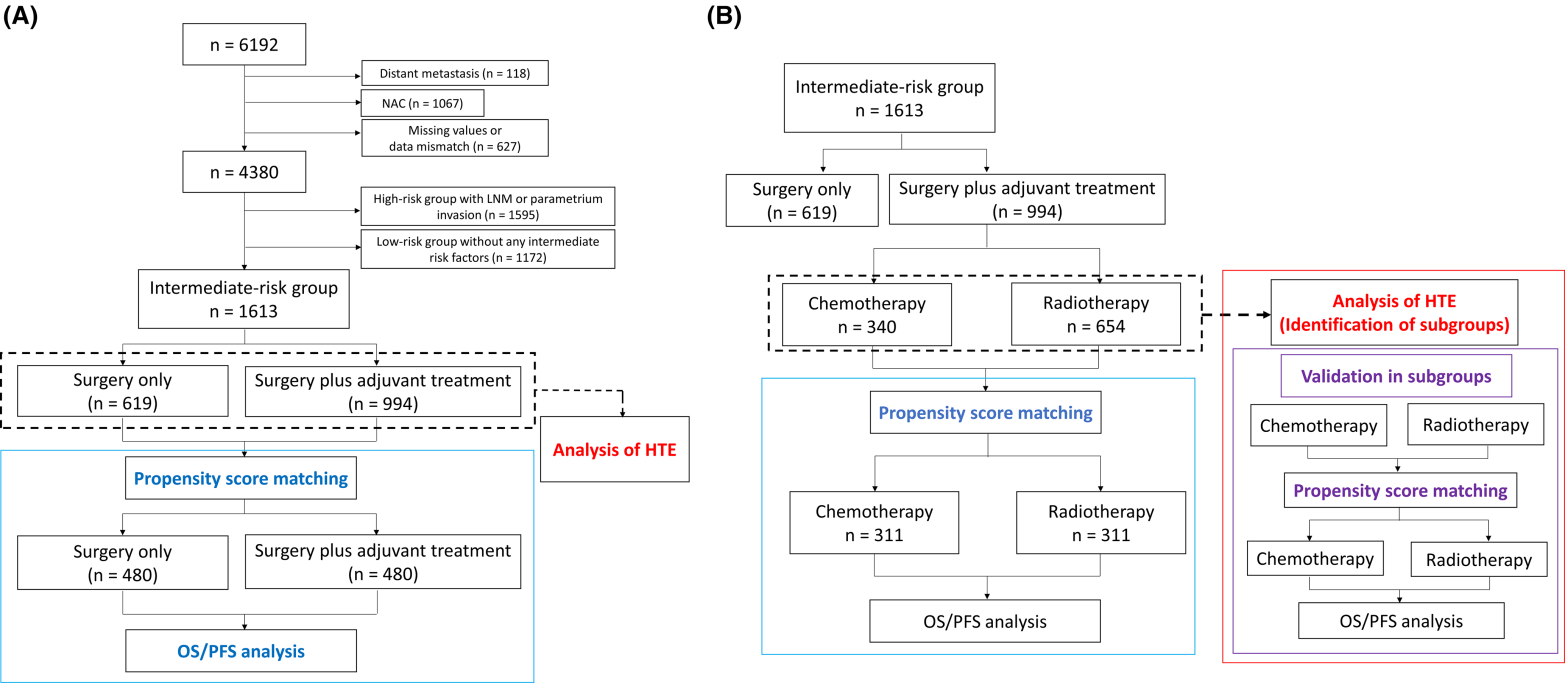
Flowchart of study selection and statistical analyses. (A) Comparison between the treatment and non‐treatment groups in patients with cervical cancer with intermediate risks. (B) Comparison between the radiation and chemotherapy groups in those in the treatment group. HTE, heterogeneous treatment effect; LNM, lymph node metastasis; NAC, neoadjuvant chemotherapy; OS, overall survival; PFS, progression‐free survival.

Patients with CC‐IR were categorized into subgroups according to the presence or absence of adjuvant therapy, namely, treatment and non‐treatment (control) (Figure 1A). Patients in the treatment subgroup were further classified based on the type of adjuvant therapy; those who underwent radiotherapy‐based therapies, including CRT and radiotherapy, were categorized into the radiotherapy subgroup (control), while those who underwent chemotherapy were categorized into the chemotherapy subgroup (Figure 1B). None of the patients in the chemotherapy subgroup received radiation‐based therapy in their primary cervical cancer treatment.

### 2.3 Statistical analyses

The Kaplan–Meier method was used in each subgroup to estimate PFS and OS following propensity score matching. Propensity scores, including the probability of receiving adjuvant treatment among patients with CC‐IR and the probability of receiving chemotherapy among the treatment groups, were estimated using logistic regression based on all clinicopathologic characteristics previously presented. Matching was performed using the “Matching” package in R (R Foundation for Statistical Computing).^22^ Standardized differences for the covariates were calculated to assess the comparability of the matched cohorts; a standardized difference <0.1 was considered to support the assumption of balance between the cohorts. The statistical difference between the curves was determined for each comparison using a log‐rank test.

HTEs were estimated using Cox proportional hazard regression models that considered interactions between treatment indicators and each covariate, as previously reported.^18^ Statistically significant coefficients of the interaction terms demonstrated the heterogeneous impact of adjuvant therapy on survival outcomes.

First, PFS and OS were compared between the treatment and non‐treatment groups using a propensity score‐matched cohort (treatment vs. non‐treatment) (Figure 1A). Second, a Cox proportional hazard regression model that considered the interaction with adjuvant therapy (treatment) was used to evaluate the HTEs of adjuvant therapy (Figure 1A).

PFS and OS were compared between the chemotherapy and radiotherapy groups to evaluate therapeutic effects according to the type of adjuvant therapy (Figure 1B). Subsequently, a Cox proportional hazard regression model that considered the interaction with adjuvant treatments was used to evaluate the HTEs of chemotherapy (treatment) compared with radiotherapy (control). After identifying subgroups in which chemotherapy was more (or less) effective than radiotherapy, background characteristics were adjusted between the chemotherapy and radiotherapy groups by propensity score matching (Figure 1B). OS and PFS were compared between the chemotherapy and radiotherapy groups in each subgroup. Statistical significance was defined as *p* < 0.05. All analyses were conducted using R (version 4.2.0).

## 3 RESULTS

### 3.1 Comparison between the treatment and non‐treatment groups

Among the 6192 patients in the original cohort, 1613 were enrolled in this analysis (Figure 1A). Among them, 619 patients did not receive any adjuvant therapy (non‐treatment group), whereas 994 patients received either radiotherapy‐based adjuvant treatments or chemotherapy (treatment group) after hysterectomy. The baseline characteristics of the patients before and after matching are presented in Table S1. Indications for adjuvant therapies were based on recurrence risk factors, including positive LVSI, large size (≥4 cm), and deep stromal invasion to the outer half. Other factors, including uterine corpus invasion and vaginal invasion, also affected the indications for adjuvant therapy. Of the 1613 patients with CC‐IR, 480 non‐treatment patients were matched with 480 treatment patients (Figure 1A). For all covariates, the absolute standardized difference was <0.1 after matching, suggesting sufficiently balanced treatment and non‐treatment groups (Table S1).

**FIGURE 2:**
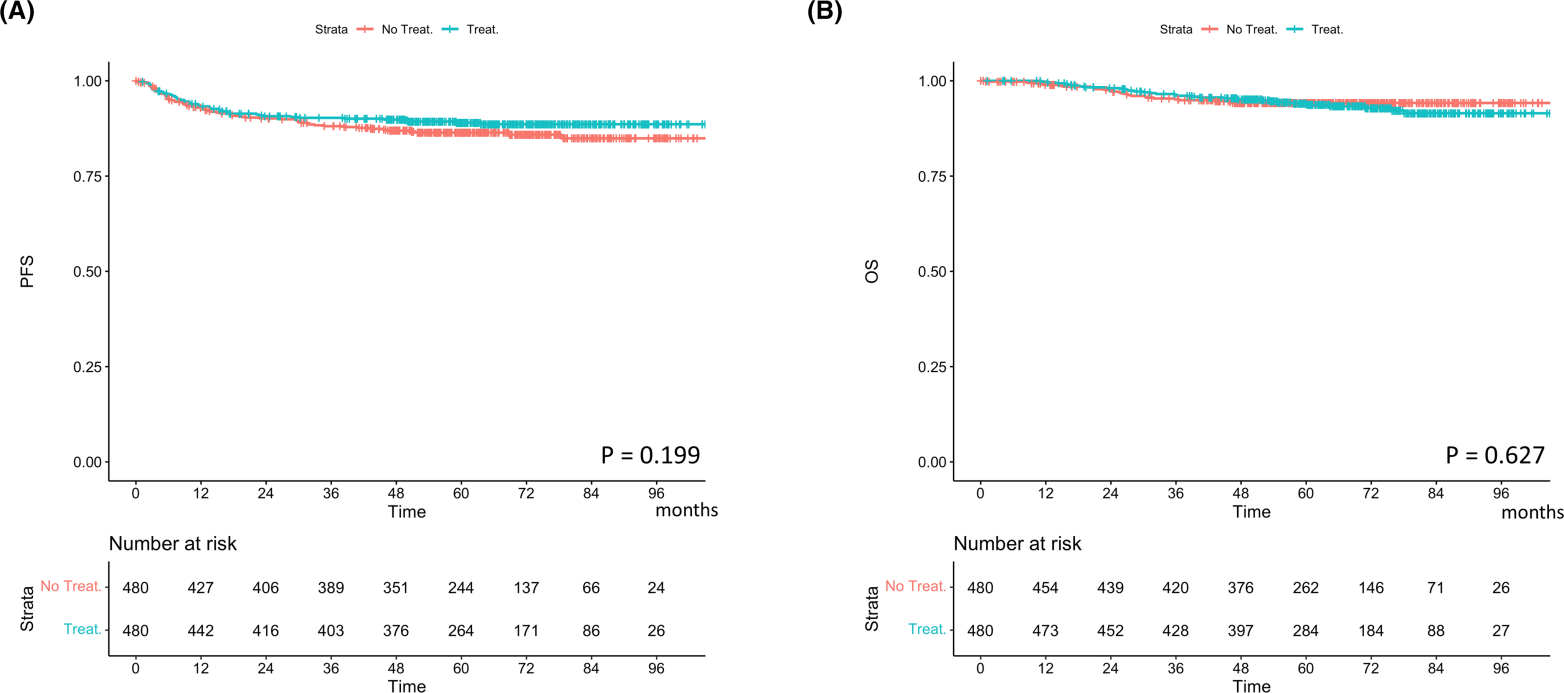
Cumulative incidence curves for (A) recurrence and (B) cervical cancer death based on the presence or absence of adjuvant treatment. OS, overall survival; PFS, progression‐free survival.

Kaplan–Meier curves based on adjuvant treatment after propensity score matching are shown in Figure 2A,B. Although patients with adjuvant treatment tended to have better PFS than those without adjuvant treatment, no significant differences were observed between the two groups: 3‐year PFS rates were 88.1% and 90.3% (*p* = 0.199, Figure 2A) and 5‐year OS rates were 94.2% and 94.0% in the non‐treatment and treatment groups, respectively (*p* = 0.627, Figure 2B).

Subsequently, we conducted a HTE analysis to identify subgroups that could have a better prognosis with adjuvant treatment (Table 1A,B). “Baseline (non‐treatment)” represents the impact of each factor on patient prognosis in the absence of adjuvant treatment (non‐treatment group), and the “interaction term” represents changes in prognosis by adding adjuvant treatment with or without each factor. Therefore, a positive (negative) exponentiated coefficient for the interaction term of a factor could be interpreted as evidence of the factor's enhancing (diminishing) effects on the impact of adjuvant treatment on OS or PFS. The “baseline” OS and PFS were better for patients with SCC than for those with non‐SCC. On the other hand, the treatment effect of adding adjuvant treatment (shown as “interaction term”) did not change with or without each factor (Table 1A,B).

**TABLE 1:** Heterogeneous treatment effect of adjuvant therapy after surgery.

Variables	Baseline (non‐treatment)			Interaction terms		
	Exp(coef)	95% CI	*p*‐Values	Exp(coef)	95% CI	*p*‐Values
A. Overall survival						
Age						
40 to <50 years	0.527	0.157–1.770	0.300	1.246	0.316–4.914	0.752
50 to <60 years	1.127	0.394–3.225	0.822	0.861	0.256–2.897	0.810
60 to <70 years	1.179	0.348–3.990	0.791	1.009	0.251–4.041	0.989
≥70 years	2.600	0.168–0.667	0.168	0.195	0.017–2.217	0.187
Histology SCC	0.426	0.193–0.942	0.035	1.365	0.545–3.419	0.506
Size						
2 to <4 cm	2.423	0.678–8.659	0.173	0.632	0.133–2.990	0.562
≥4 cm	1.510	0.338–6.739	0.588	1.764	0.308–10.09	0.523
LVSI positive	1.412	0.586–3.401	0.441	1.998	0.648–6.158	0.227
Cervical stromal invasion ≥1/2	1.555	0.632–3.821	0.335	0.833	0.288–2.408	0.736
Uterine body invasion	1.981	0.634–6.189	0.239	0.556	0.149–2.070	0.381
Vaginal invasion	1.993	0.774–5.134	0.152	0.706	0.241–2.063	0.524
High‐volume center	0.578	0.238–1.401	0.225	0.289	0.476–3.742	0.582
B. Progression‐free survival						
Age						
40 to <50 years	0.615	0.306–1.235	0.172	1.923	0.836–4.422	0.124
50 to <60 years	1.101	0.567–2.137	0.776	0.935	0.411–2.124	0.872
60 to <70 years	1.765	0.887–3.515	0.106	0.692	0.288–1.659	0.409
≥70 years	1.699	0.628–4.598	0.297	0.537	0.114–2.536	0.433
Histology SCC	0.534	0.327–0.873	0.012	1.054	0.579–1.920	0.863
Size						
2 to <4 cm	2.516	1.153–5.489	0.020	0.738	0.262–2.078	0.565
≥ 4 cm	2.893	1.230–6.805	0.015	0.789	0.262–2.375	0.673
LVSI positive	1.345	0.309–0.939	0.2899	1.266	0.634–2.529	0.504
Cervical stromal invasion ≥1/2	1.156	0.688–1.943	0.583	0.855	0.444–1.644	0.638
Uterine body invasion	1.389	0.635–3.042	0.411	0.992	0.396–2.487	0.987
Vaginal invasion	1.828	1.009–3.309	0.046	0.813	0.403–1.640	0.563
High‐volume center	0.539	0.309–0.939	0.029	1.066	0.529–2.145	0.859

*Note*: A. Overall survival; B. Progression‐free survival. “Baseline (non‐treatment)” represents the impact of each factor on patient prognosis in the absence of adjuvant treatment (non‐treatment group), and the “interaction term” represents changes in prognosis by adding adjuvant treatment with or without each factor. Therefore, a positive (negative) exponentiated coefficient for the interaction term of a factor could be interpreted as evidence of the factor's enhancing (diminishing) effects on the impact of adjuvant treatment on overall survival.

Abbreviations: CI, confidence interval; Exp(coef), exponentiated coefficient; LVSI, lymphovascular space invasion; SCC, squamous cell carcinoma.

Next, we compared the rates and types of adjuvant therapy based on the type of institution. No difference was found between high‐volume and non‐high‐volume centers in the rates or types of adjuvant therapies (Figure S1). Among the high‐volume centers, the frequency of adjuvant therapy differed between the institutions (Figure S2); however, at least 30% of the patients received adjuvant therapy in all institutions.

As previous studies have demonstrated that the prognosis of patients with CC‐IR worsens according to the number of recurrence risk factors, we subsequently analyzed the frequency of adjuvant therapy based on the number of recurrence risk factors. The frequency of adjuvant therapy increased with the number of recurrence risk factors: 43.5%, 74.8%, and 86.2% in those with one, two, and three risk factors, respectively (Figure S3). In the matched cohort, 61% of the patients had one risk factor (tumor size, interstitial invasion, or LVSI), whereas the other 39% had two or more risk factors (Figure S4A,B).

### 3.2 Comparison between the chemotherapy and radiotherapy groups in the treatment group

Because most patients with more than two recurrent risk factors received adjuvant therapy, we next focused on the effect of chemotherapy on CC‐IR compared with radiotherapy‐based treatment. Of the 994 patients with CC‐IR in the treatment group, 654 and 340 received radiotherapy and chemotherapy, respectively. More than half of the regimens were taxane–platinum combinations, such as paclitaxel plus carboplatin or cisplatin (Table S2). Patient background characteristics were compared between the chemotherapy and radiotherapy groups (Table S3). Patients with non‐SCC histology tended to receive adjuvant chemotherapy rather than radiotherapy. In contrast, those with larger tumor sizes, uterine body invasion, and vaginal invasion tended to receive radiotherapy rather than chemotherapy.

Of the 994 patients in the treatment group, 311 patients in the chemotherapy group were matched with 311 patients in the radiotherapy group (Figure 1B). For all covariates, the absolute standardized difference was <0.1 after matching, suggesting sufficiently balanced chemotherapy and radiotherapy groups (Table S3). In both groups, 62% of the patients had two or more risk factors (Figure S5A,B); this proportion was significantly higher than that in the non‐treatment and treatment groups (39%).

**FIGURE 3:**
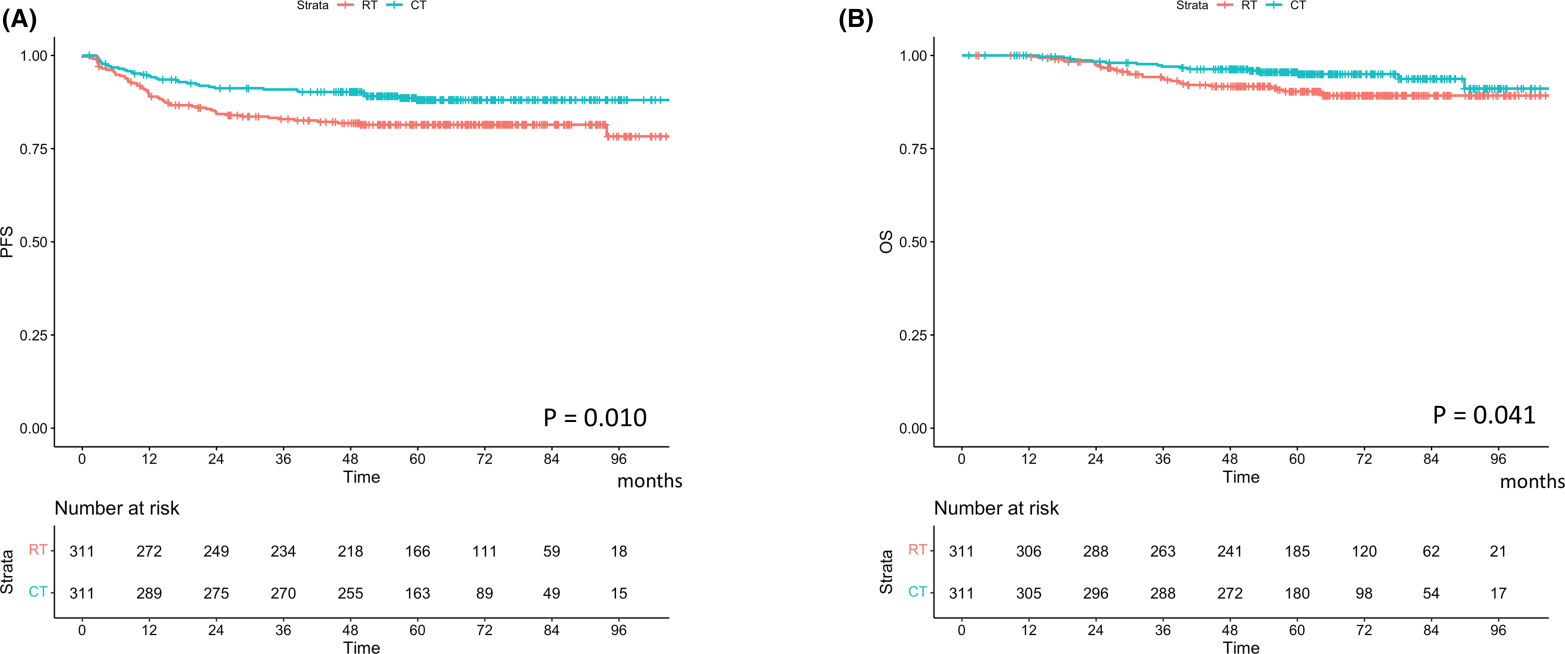
Cumulative incidence curves for (A) recurrence and (B) cervical cancer death based on the types of adjuvant therapy. OS, overall survival; PFS, progression‐free survival.

Kaplan–Meier curves of PFS and OS of patients who received chemotherapy and radiotherapy are shown in Figure 3A,B. Patients in the chemotherapy group had better PFS and OS than those in the radiotherapy group: 3‐year PFS rates were 90.9% and 82.9%, respectively (*p* = 0.010), and 5‐year OS rates were 95.5% and 90.3%, respectively (*p* = 0.041).

Subsequently, we conducted an HTE analysis to identify subgroups that could have a better prognosis with adjuvant chemotherapy than with adjuvant radiotherapy‐based treatments (Table 2A,B). “Baseline (radiation)” represents the impact of each factor on the prognosis of patients with adjuvant radiotherapy, and the “interaction term” represents changes in prognosis with adjuvant chemotherapy (compared with adjuvant radiotherapy) with or without each factor. The “baseline” PFS was better for patients with SCC or small tumors than for those with non‐SCC or large tumors. However, only tumor size significantly affected the treatment effect of adjuvant chemotherapy; the treatment effect of adjuvant chemotherapy was higher in patients with large tumors than that of adjuvant radiotherapy.

**TABLE 2:** Heterogeneous treatment effect of adjuvant chemotherapy.

Variables	Baseline (radiation)			Interaction terms		
	Exp(coef)	95% CI	*p*‐Values	Exp(coef)	95% CI	*p*‐Values
A. Overall survival						
Age						
40 to <50 years	0.553	0.259–1.182	0.127	1.684	0.383–7.415	0.491
50 to <60 years	0.886	0.435–1.808	0.74	1.346	0.33–5.482	0.678
60 to <70 years	0.98	0.455–2.11	0.959	1.8	0.387–8.38	0.454
≥70 years	0.585	0.077–4.445	0.604	1.34 × 10^−6^	n.c	0.995
Histology SCC	0.581	0.33–1.023	0.06	0.726	0.236–2.233	0.576
Size						
2 to <4 cm	2.023	0.596–6.86	0.258	0.434	0.069–2.734	0.374
≥4 cm	2.962	0.869–10.1	0.083	0.831	0.128–5.411	0.846
LVSI positive	2.16	1.009–4.623	0.047	4.239	0.484–37.13	0.192
Cervical stromal invasion ≥1/2	1.255	0.635–2.481	0.513	1.116	0.323–3.856	0.862
Uterine body invasion	0.1.344	0.646–2.797	0.429	0.419	0.074–2.368	0.325
Vaginal invasion	1.471	0.833–2.596	0.184	0.527	0.129–2.142	0.37
High‐volume center	0.766	0.425–1.379	0.374	0.703	0.175–2.825	0.619
B. Progression‐free survival						
Age						
40 to <50 years	1.022	0.59–1.768	0.939	1.484	0.549–4.012	0.436
50 to <60 years	0.981	0.547–1.76	0.949	1.109	0.384–3.204	0.848
60 to <70 years	1.289	0.698–2.38	0.417	0.614	0.163–2.317	0.472
≥ 70 years	0.73	0.172–3.104	0.67	2.63	0.193–35.85	0.468
Histology SCC	0.509	0.335–0.773	0.002	0.973	0.439–2.158	0.947
Size						
2 to <4 cm	3.799	1.166–12.38	0.027	0.214	0.048–0.946	0.042
≥ 4 cm	4.631	1.411–15.2	0.011	0.207	0.044–0.985	0.048
LVSI positive	1.356	0.829–2.217	0.226	2.727	0.848–8.774	0.092
Cervical stromal invasion ≥1/2	0.918	0.566–1.489	0.729	1.42	0.593–3.401	0.431
Uterine body invasion	1.6	0.943–2.715	0.082	0.384	0.098–1.515	0.172
Vaginal invasion	1.479	0.966–2.263	0.072	0.633	0.236–1.693	0.362
High‐volume center	0.649	0.407–1.035	0.07	0.37	0.102–1.337	0.129

*Note*: A. Overall survival; B. Progression‐free survival. “Baseline (radiation)” represents the impact of each factor on the prognosis of patients with adjuvant radiotherapy, and the “interaction term” represents changes in prognosis with adjuvant chemotherapy (compared with adjuvant radiotherapy) with or without each factor. Therefore, a positive (negative) exponentiated coefficient for the interaction term of a factor could be interpreted as evidence of the factor's enhancing (diminishing) effects on the impact of adjuvant chemotherapy on overall survival and progression‐free survival.

Abbreviations: CI, confidence interval; Exp(coef), exponentiated coefficient; LVSI, lymphovascular space invasion; SCC, squamous cell carcinoma.

**FIGURE 4:**
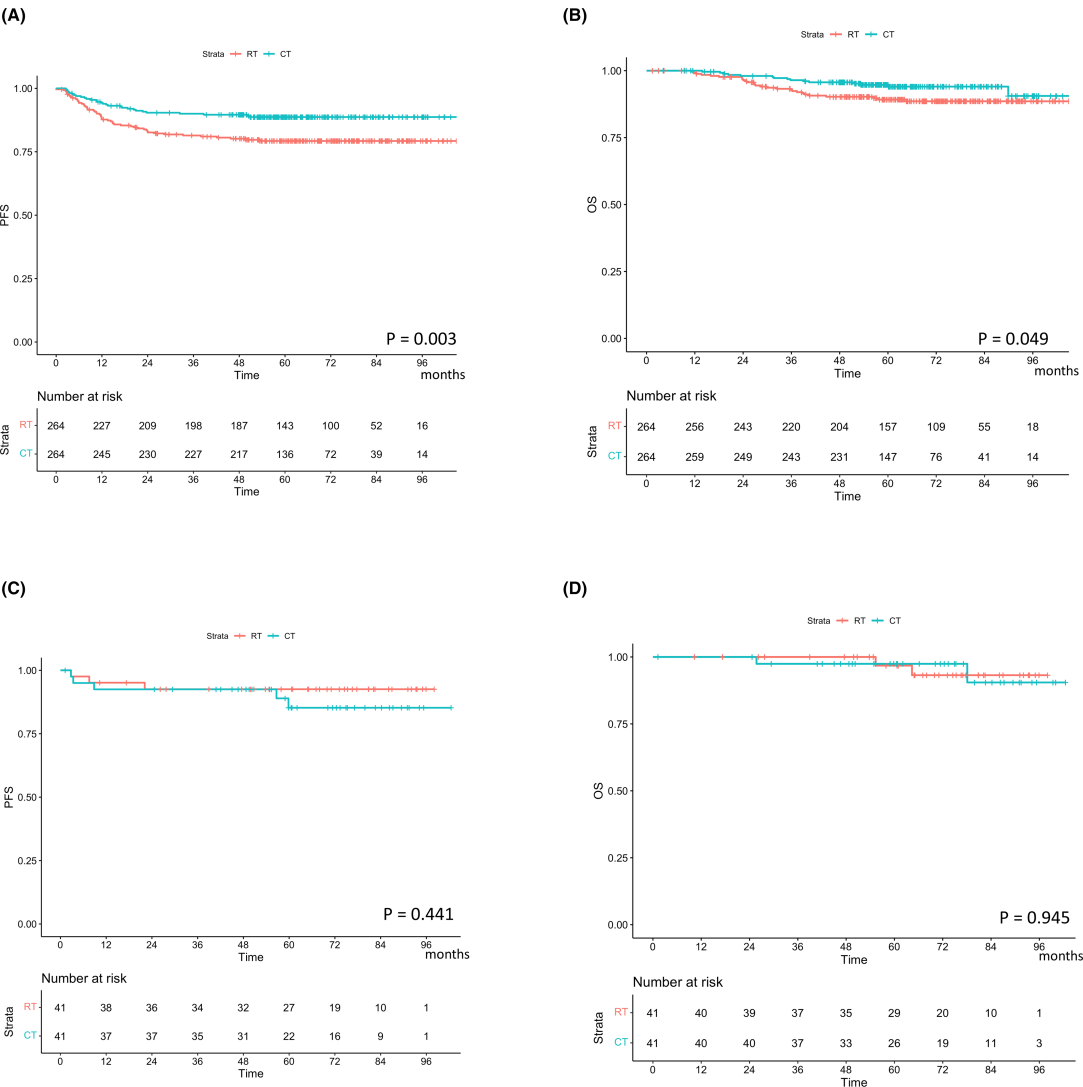
Cumulative incidence curves for recurrence and cervical cancer death based on the types of adjuvant therapy according to tumor sizes. (A) Recurrence in patients with large tumors (≥2 cm); (B) death from cervical cancer in patients with large tumors (≥2 cm); (C) recurrence in patients with small tumors (<2 cm); (D) death from cervical cancer in patients with small tumors (<2 cm). OS, overall survival; PFS, progression‐free survival.

Subgroup analysis was conducted after propensity score matching in each subgroup to validate the treatment effect of chemotherapy compared with that of radiotherapy (Figure S6A,B). The background characteristics of patients with small tumor sizes (<2 cm) and large tumor sizes (≥2 cm) are summarized in Table S4A,B. After propensity score matching in each subgroup, the absolute standardized difference for all covariates except for age distribution was <0.1 after matching. In patients with large tumor sizes (≥2 cm), those who received chemotherapy had better PFS and OS than those who received radiotherapy‐based treatments: 3‐year PFS rates were 90.0% and 81.4% in the chemotherapy and radiotherapy‐based therapy groups, respectively (*p* = 0.003, Figure 4A), and 5‐year OS rates were 94.7% and 89.2%, respectively (*p* = 0.049, Figure 4B). In contrast, no difference was observed between the two groups in patients with small tumors (<2 cm): 3‐year PFS rates were 92.5% and 92.6% in the chemotherapy and radiotherapy‐based therapy groups, respectively (*p* = 0.441, Figure 4C), and 5‐year OS rates were 97.4% and 96.8%, respectively (*p* = 0.945, Figure 4D).

## 4 DISCUSSION

In this study, we analyzed the significance of adjuvant therapies for patients with CC‐IR using a nationwide cohort database. Due to adjuvant therapy's selection bias, it has been difficult to perform unbiased analyses using single‐center data. However, because of the differences in treatment selection (for both indications and types of adjuvant therapy) between facilities, using a nationwide cohort database decreases the bias.

In the first analysis, we compared survival outcomes between patients with CC‐IR with and without adjuvant treatment and revealed that adjuvant treatment had no significant treatment effects on survival. The treatment and non‐treatment groups had similar 3‐year PFS rates (88.1% and 90.3%, respectively), which are similar to the rates reported previously in the treatment group.^7^ There are two possible reasons for the good survival rate even in the non‐treatment group. One reason is that relatively low‐risk patients were included in the propensity score‐matched cohort (treatment vs. non‐treatment); approximately 60% of the patients had only one risk factor. The other reason relates to the surgical procedure for radical hysterectomy in Japan. Okabayashi radical hysterectomy is widely used in Japan, which corresponds to Type III hysterectomy of the Gynecologic Cancer Group, and has sufficient efficacy for local disease control.^23^

The indications and the type of adjuvant therapy depend on the institutions. Therefore, we could compare the treatment effects between the radiotherapy and chemotherapy groups. Our results indicated that patients with CC‐IR who underwent adjuvant chemotherapy had a better prognosis than those who received adjuvant radiotherapy, with 3‐year PFS rates of 90.9% and 82.9%, respectively, which is better than those of previous reports.^7^, ^12^, ^14^ Furthermore, we investigated the HTEs of adjuvant chemotherapy compared with adjuvant radiotherapy and identified tumor size as the most influential factor for differential treatment effects between adjuvant chemotherapy and radiotherapy. Adjuvant chemotherapy was more effective than adjuvant radiotherapy in patients with large tumors. Patients with tumors measuring <2 cm had a good prognosis, regardless of the type of adjuvant therapy. Considering that approximately 80% of the patients with small tumors (<2 cm) had only one recurrent risk factor (Figure S7A,B), the treatment effects of adjuvant therapy (regardless of the type of adjuvant therapy) may be limited in these patients.

Notably, adjuvant chemotherapy prolonged OS and improved PFS in patients with cervical cancer. Only definitive radiotherapy‐ and surgical‐based therapies are recognized as definitive therapeutics for cervical cancer,^4^ and chemotherapy‐based therapies are not as definitive as radiotherapy; however, paclitaxel plus platinum‐based chemotherapy regimens have sufficient efficacy with a response rate of 30%–62% for advanced or recurrent cervical cancers.^24^, ^25^, ^26^, ^27^ Therefore, chemotherapy might be an alternative adjuvant therapy to radiotherapy for patients with CC‐IR who underwent Okabayashi radical hysterectomy.

This study had some limitations. First, even when using propensity score matching, we were uncertain about the effect of unobserved factors on disease recurrence or OS. Particularly, information regarding patients' general condition that could affect both the indication for adjuvant therapy and survival, including their performance status and complications, may have improved comparability between the groups. Therefore, a prospective randomized controlled study is warranted to validate the results of this study. Second, because only approximately one fifth of patients with two or more recurrent risk factors were in the non‐treatment group, most of the patients in the matched analysis had only one risk factor, and a limited number of those with two or more risk factors were included. Therefore, indications for adjuvant therapy should be carefully determined for patients with multiple risk factors. However, this study is still important because it confirmed that adjuvant therapy has no treatment effects on PFS and OS in patients with CC‐IR (especially those with one risk factor) who are treated at Japanese hospitals. Third, this was a retrospective study with missing data. Some survival events could have been underestimated because of insufficient prognostic follow‐up and short observational periods.

In conclusion, adjuvant therapy had no therapeutic effect for some patients; therefore, adjuvant therapy is optional for patients with CC‐IR. However, these results should be applied with caution to patients with multiple risk factors. Regarding the type of adjuvant therapy, chemotherapy may be recommended for patients with CC‐IR, particularly those with large tumor sizes. Further prospective randomized controlled studies are warranted to confirm these results.

## AUTHOR CONTRIBUTIONS

**Ayumi Taguchi:** Conceptualization (lead); data curation (equal); investigation (equal); visualization (equal); writing – original draft (lead). **Kosuke Kato:** Data curation (equal); formal analysis (lead); investigation (equal); visualization (equal). **Konan Hara:** Conceptualization (equal); methodology (lead); supervision (equal); writing – review and editing (equal). **Akiko Furusawa:** Project administration (equal); writing – review and editing (equal). **Yujiro Nakajima:** Data curation (supporting); formal analysis (supporting). **Chihiro Ishizawa:** Writing – review and editing (equal). **Michihiro Tanikawa:** Writing – review and editing (equal). **Kenbun Sone:** Writing – review and editing (equal). **Mayuyo Mori:** Supervision (supporting); writing – review and editing (equal). **Muneaki Shimada:** Data curation (equal); supervision (equal); writing – review and editing (equal). **Aikou Okamoto:** Supervision (equal). **Munetaka Takekuma:** Supervision (equal); writing – review and editing (equal).

## FUNDING INFORMATION

No funding.

## CONFLICT OF INTEREST STATEMENT

The authors declare that they have no conflict of interest.

## ETHICS STATEMENT

Institutional review board approval for this study was obtained from the University of Tokyo (approval number: 2021078NI‐2) and Tokyo Metropolitan Komagome Hospital (approval number: 2749).

## PATIENT CONSENT

The institutional review board granted an opt‐out recruitment approach and waived the need for obtaining written informed consent. We adhered to the Declaration of Helsinki.

## Supporting information


Figures S1–S7.

Tables S1–S4.

## Data Availability

The data that support the findings of this study are available from the corresponding author, MT, upon reasonable request.
