# Supplementary material for: Heterogeneous treatment effects of adjuvant therapy for patients with cervical cancer in the intermediate‐risk group
Source: Cancer Med. 2023 Aug 16;12(18):18557–67. doi: 10.1002/cam4.6460 (PMC10557871; doi:10.1002/cam4.6460)
Supplement: Supplementary file 1 — Figures S1–S7. [file CAM4-12-18557-s002.pptx]

## Slide 1
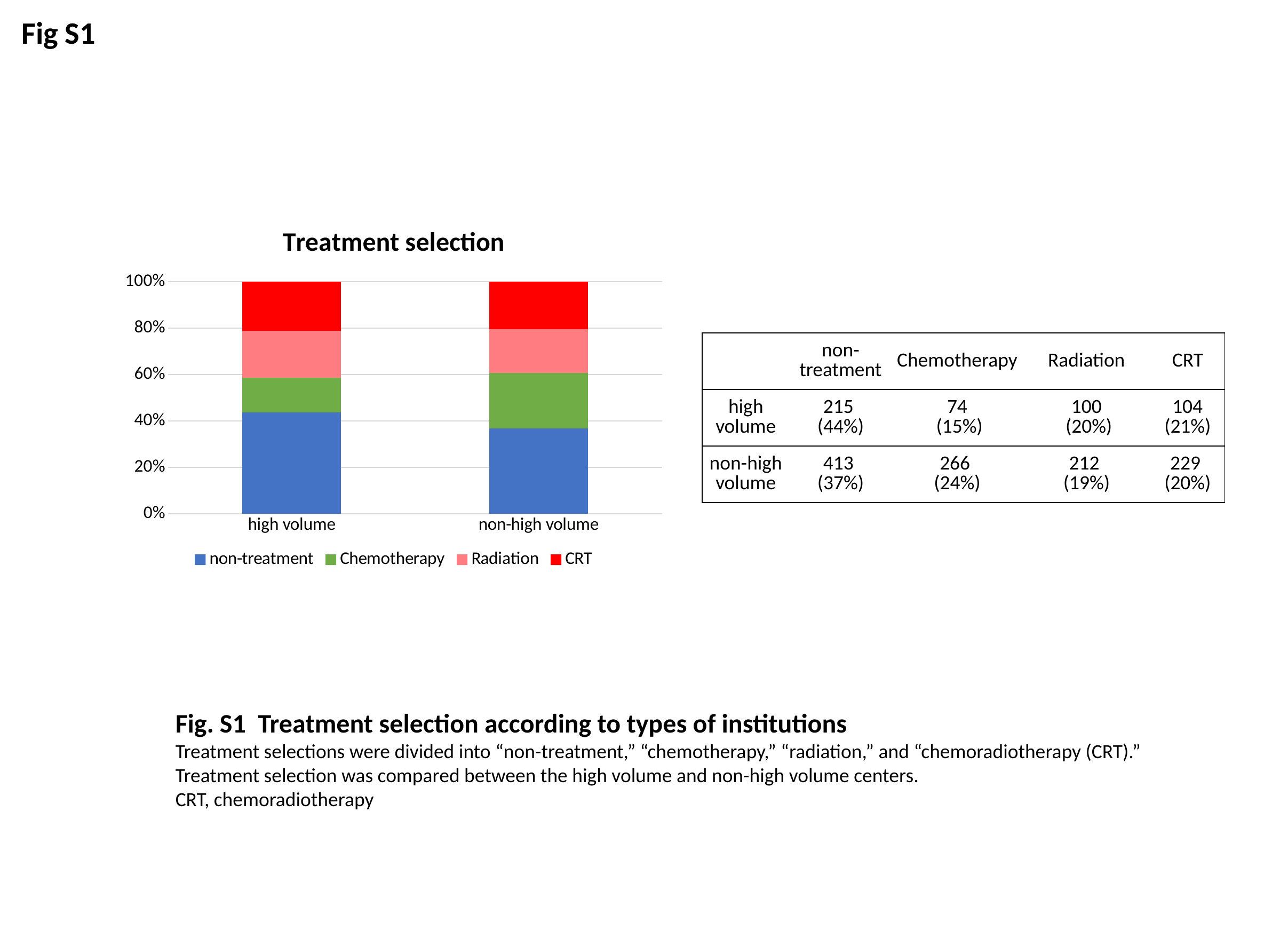

Fig S1
### Chart: Treatment selection
| Category | non-treatment | Chemotherapy | Radiation | CRT |
|---|---|---|---|---|
| high volume | 215.0 | 74.0 | 100.0 | 104.0 |
| non-high volume | 413.0 | 266.0 | 212.0 | 229.0 || | non-treatment | Chemotherapy | Radiation | CRT |
| --- | --- | --- | --- | --- |
| high volume | 215 (44%) | 74 (15%) | 100 (20%) | 104 (21%) |
| non-high volume | 413 (37%) | 266 (24%) | 212 (19%) | 229 (20%) |
Fig. S1 Treatment selection according to types of institutions
Treatment selections were divided into “non-treatment,” “chemotherapy,” “radiation,” and “chemoradiotherapy (CRT).” Treatment selection was compared between the high volume and non-high volume centers.
CRT, chemoradiotherapy

## Slide 2
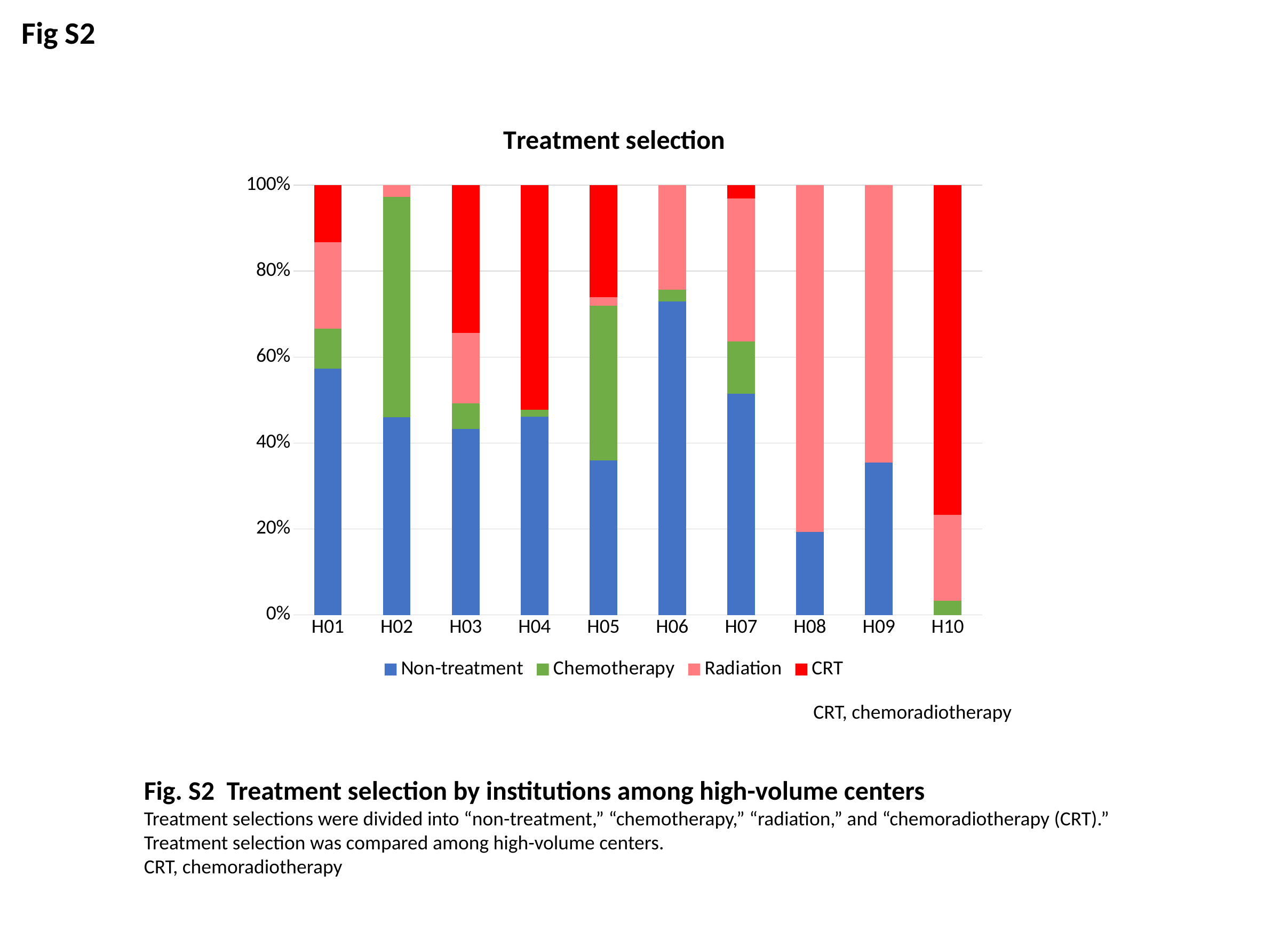

Fig S2
### Chart: Treatment selection
| Category | Non-treatment | Chemotherapy | Radiation | CRT |
|---|---|---|---|---|
| H01 | 43.0 | 7.0 | 15.0 | 10.0 |
| H02 | 34.0 | 38.0 | 2.0 | 0.0 |
| H03 | 29.0 | 4.0 | 11.0 | 23.0 |
| H04 | 30.0 | 1.0 | 0.0 | 34.0 |
| H05 | 18.0 | 18.0 | 1.0 | 13.0 |
| H06 | 27.0 | 1.0 | 9.0 | 0.0 |
| H07 | 17.0 | 4.0 | 11.0 | 1.0 |
| H08 | 6.0 | 0.0 | 25.0 | 0.0 |
| H09 | 11.0 | 0.0 | 20.0 | 0.0 |
| H10 | 0.0 | 1.0 | 6.0 | 23.0 |CRT, chemoradiotherapy
Fig. S2 Treatment selection by institutions among high-volume centers
Treatment selections were divided into “non-treatment,” “chemotherapy,” “radiation,” and “chemoradiotherapy (CRT).” Treatment selection was compared among high-volume centers.
CRT, chemoradiotherapy

## Slide 3
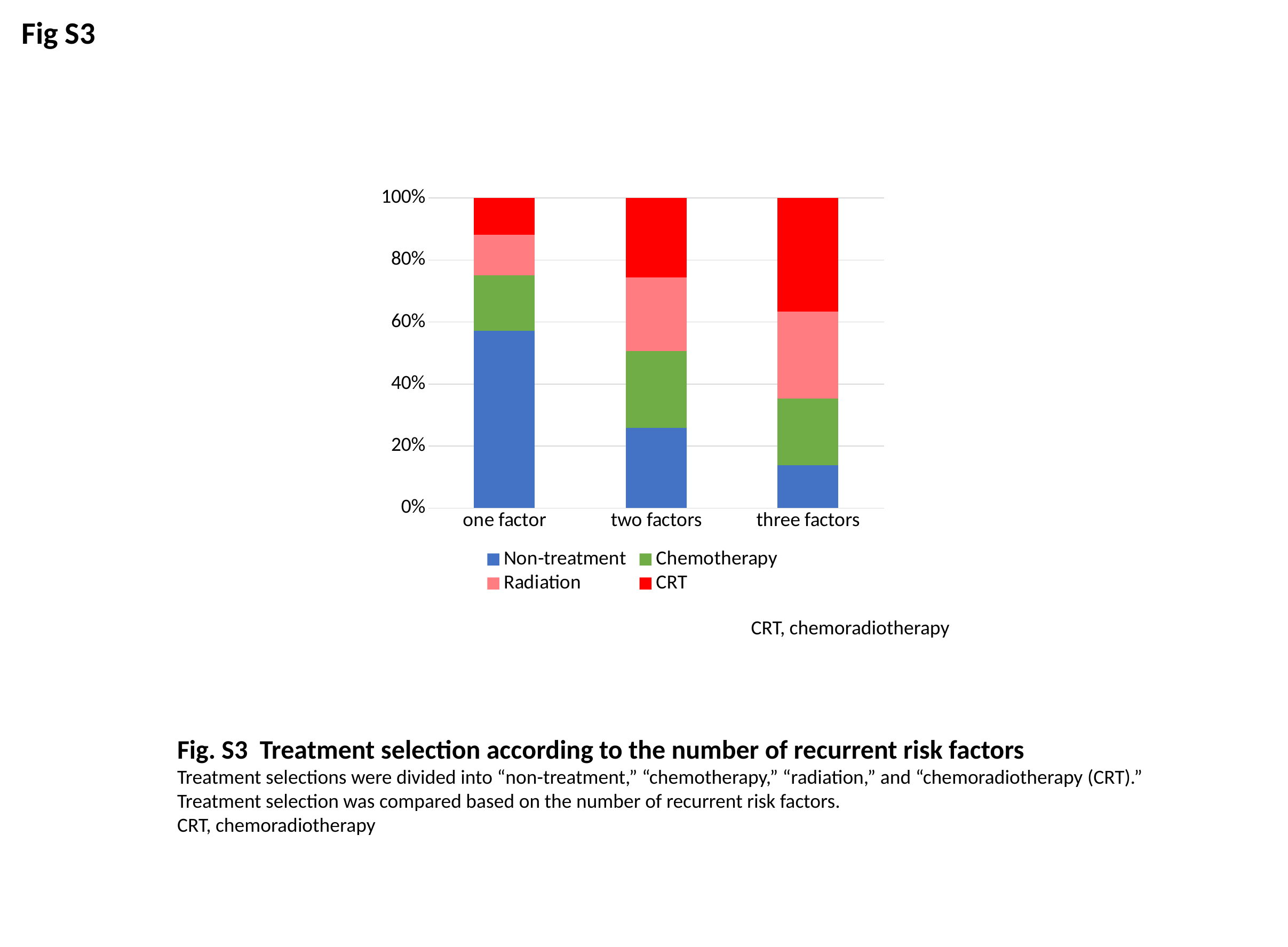

Fig S3
### Chart
| Category | Non-treatment | Chemotherapy | Radiation | CRT |
|---|---|---|---|---|
| one factor | 437.0 | 137.0 | 101.0 | 90.0 |
| two factors | 159.0 | 153.0 | 146.0 | 158.0 |
| three factors | 32.0 | 50.0 | 65.0 | 85.0 |CRT, chemoradiotherapy
Fig. S3 Treatment selection according to the number of recurrent risk factors
Treatment selections were divided into “non-treatment,” “chemotherapy,” “radiation,” and “chemoradiotherapy (CRT).” Treatment selection was compared based on the number of recurrent risk factors.
CRT, chemoradiotherapy

## Slide 4
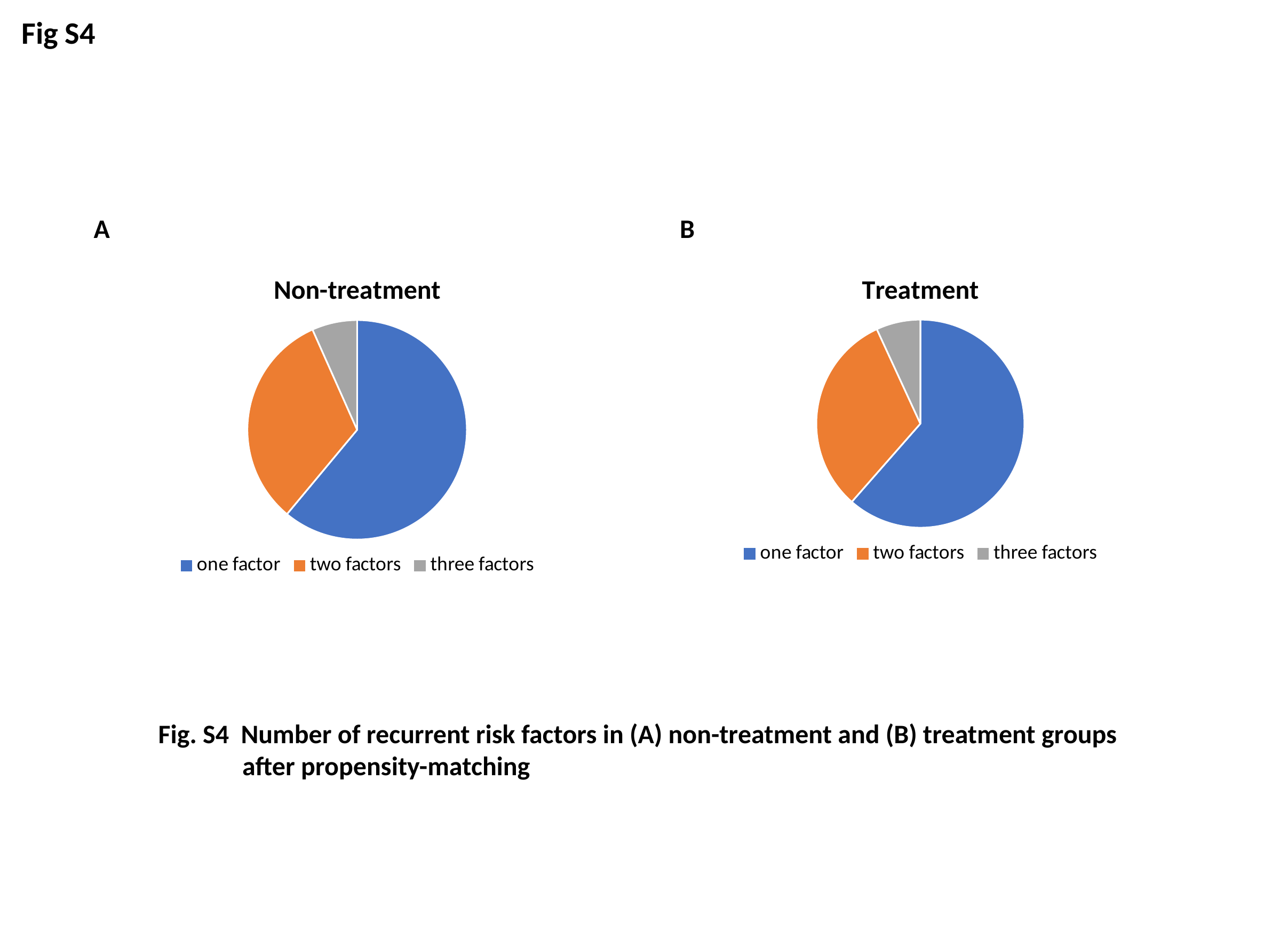

Fig S4
B
A
### Chart:
| Category | Non-treatment |
|---|---|
| one factor | 293.0 |
| two factors | 155.0 |
| three factors | 32.0 |
### Chart:
| Category | Treatment |
|---|---|
| one factor | 295.0 |
| two factors | 152.0 |
| three factors | 33.0 |Fig. S4 Number of recurrent risk factors in (A) non-treatment and (B) treatment groups
 after propensity-matching

## Slide 5
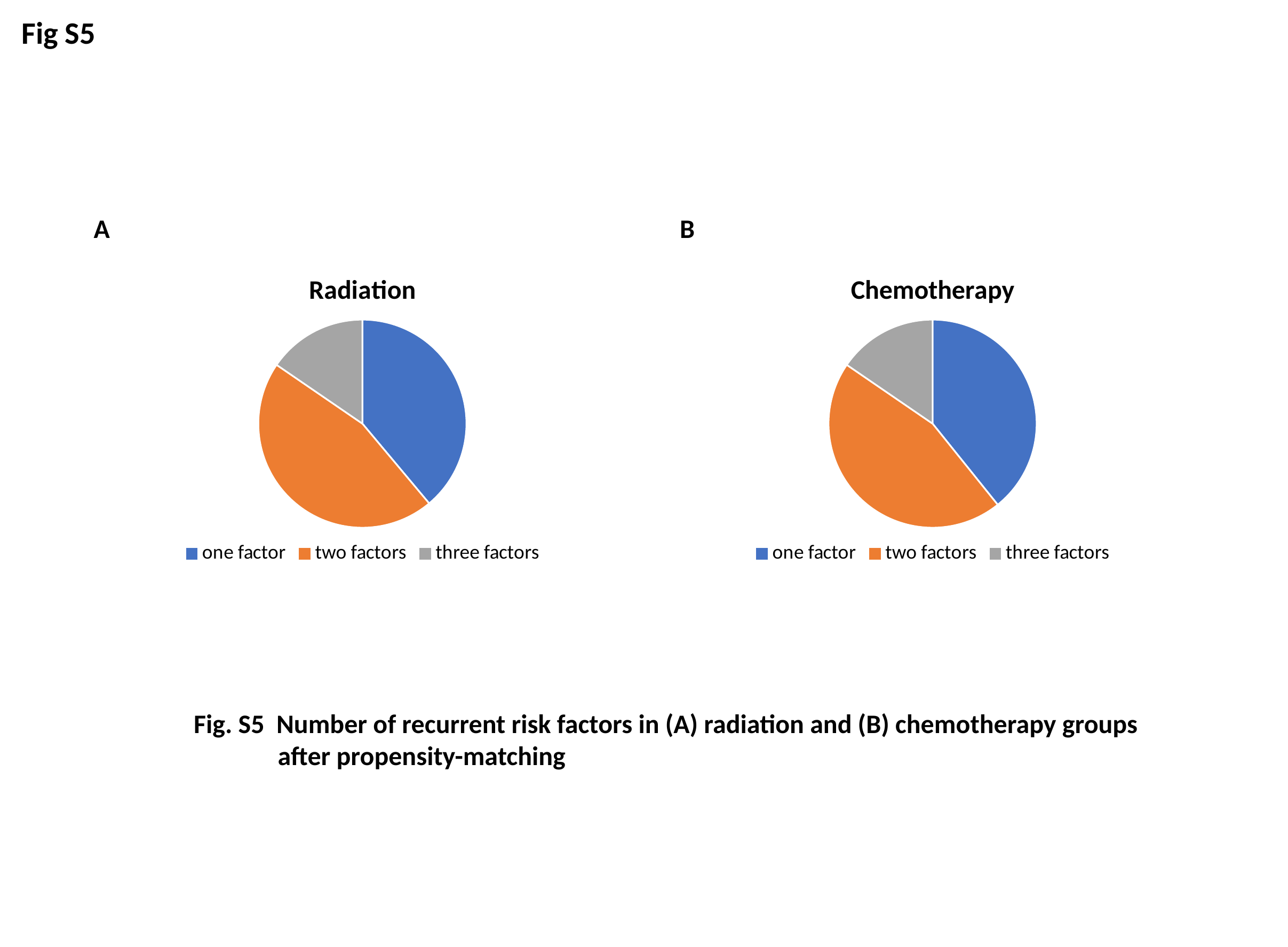

Fig S5
B
A
### Chart: Radiation
| Category | RT |
|---|---|
| one factor | 121.0 |
| two factors | 142.0 |
| three factors | 48.0 |
### Chart: Chemotherapy
| Category | CT |
|---|---|
| one factor | 122.0 |
| two factors | 141.0 |
| three factors | 48.0 |Fig. S5 Number of recurrent risk factors in (A) radiation and (B) chemotherapy groups
 after propensity-matching

## Slide 6
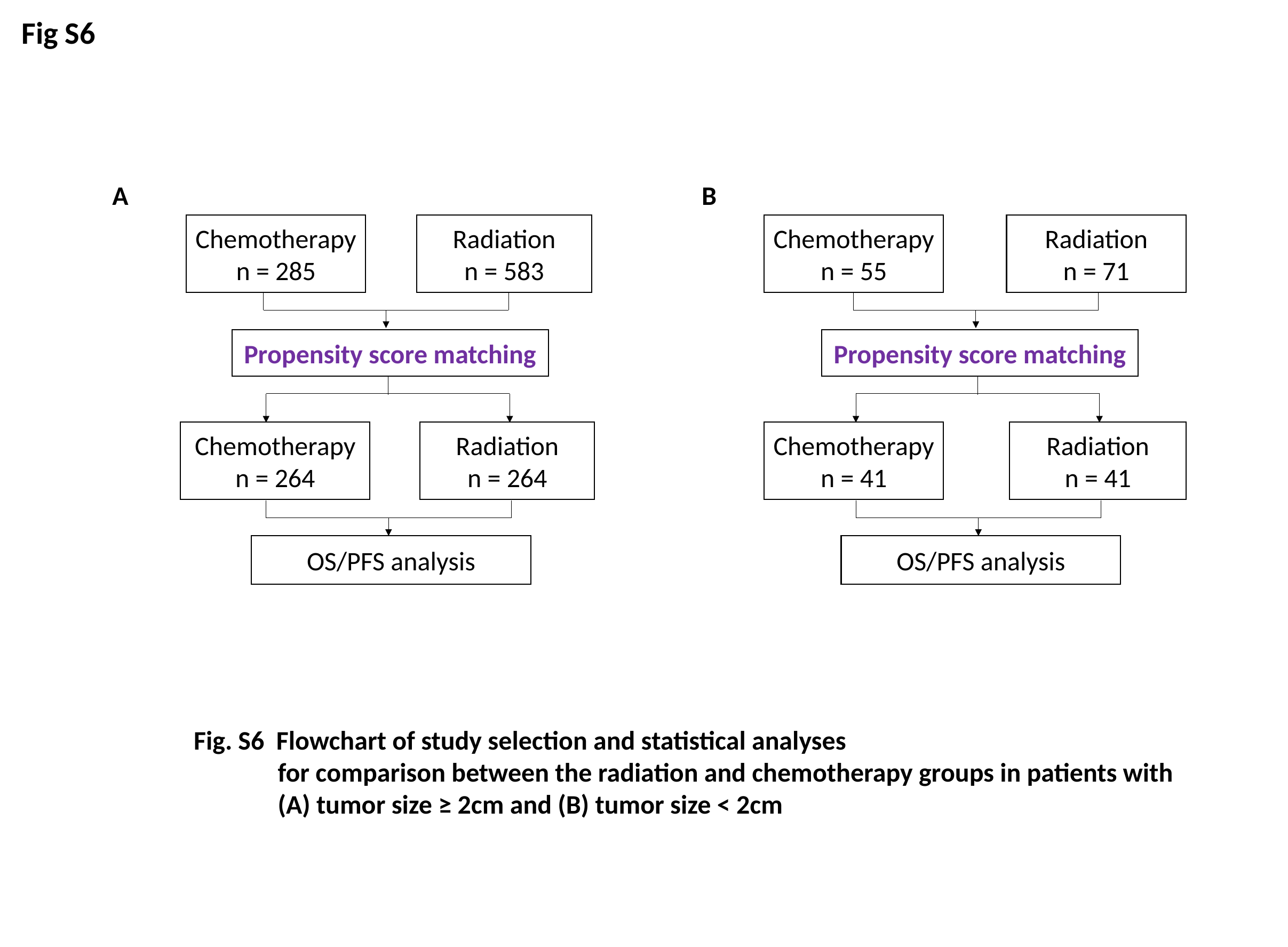

Fig S6
B
A
Chemotherapy
n = 285
Radiation
n = 583
Chemotherapy
n = 55
Radiation
n = 71
Propensity score matching
Propensity score matching
Chemotherapy
n = 264
Chemotherapy
n = 41
Radiation
n = 264
Radiation
n = 41
OS/PFS analysis
OS/PFS analysis
Fig. S6 Flowchart of study selection and statistical analyses
 for comparison between the radiation and chemotherapy groups in patients with
 (A) tumor size ≥ 2cm and (B) tumor size < 2cm

## Slide 7
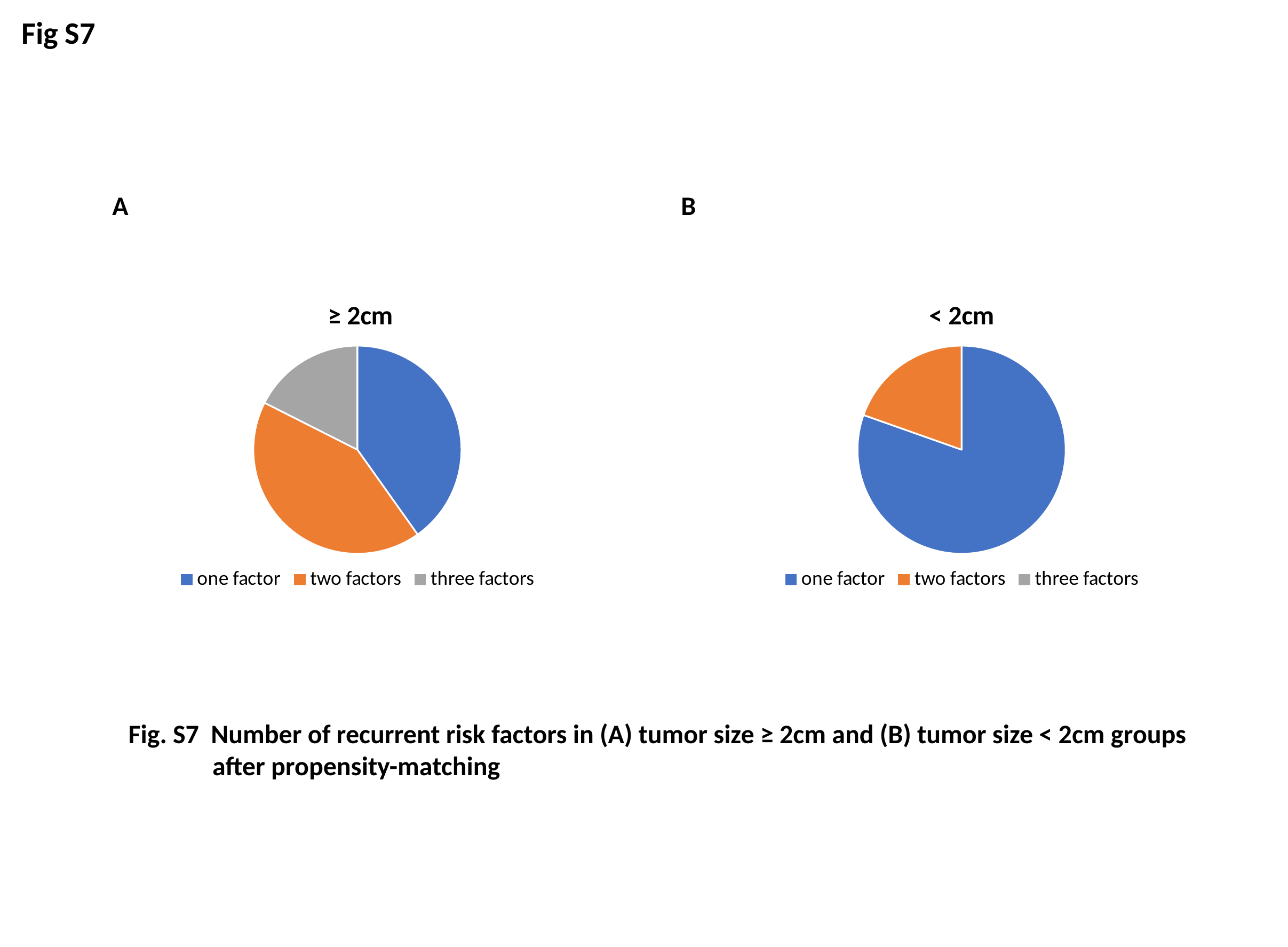

Fig S7
A
B
### Chart:
| Category | ≥ 2cm |
|---|---|
| one factor | 531.0 |
| two factors | 559.0 |
| three factors | 232.0 |
### Chart:
| Category | < 2cm |
|---|---|
| one factor | 234.0 |
| two factors | 57.0 |
| three factors | 0.0 |Fig. S7 Number of recurrent risk factors in (A) tumor size ≥ 2cm and (B) tumor size < 2cm groups
 after propensity-matching
