# Supplementary material for: Heterogeneous treatment effects of adjuvant therapy for patients with cervical cancer in the intermediate‐risk group
Source: Cancer Med. 2023 Aug 16;12(18):18557–67. doi: 10.1002/cam4.6460 (PMC10557871; doi:10.1002/cam4.6460)
Supplement: Supplementary file 2 — Tables S1–S4. [file CAM4-12-18557-s001.docx]

Table S1 Patients’ characteristics according to adjuvant treatment

|  | Before propensity score matching | | | After propensity score matching | | |
| --- | --- | --- | --- | --- | --- | --- |
|  | Non-treatment  n = 619 | Treatment  n = 994 | Standardized differences | Non-treatment  n = 480 | Treatment  n = 480 | Standardized differences |
| Age, mean  (Standard deviation) | 46.3 (12) | 48.0 (12.2) | 0.137 | 47.2 (12.0) | 46.8 (12.4) | -0.032 |
| Histology SCC | 437 (70.1) | 651 (65.5) | -0.100 | 329 (68.5) | 323 (67.3) | -0.027 |
| Size |  |  |  |  |  |  |
| 2 to <4 cm | 320 (51.7) | 492 (49.5) | -0.044 | 278 (57.9) | 274 (57.1) | -0.017 |
| ≥4 cm | 134 (21.6) | 376 (37.8) | 0.360 | 118 (24.6) | 122 (25.4) | 0.019 |
| LVSI positive | 402 (64.9) | 747 (75.2) | 0.224 | 303 (63.1) | 301 (62.7) | -0.0086 |
| Cervical stromal invasion ≥1/2 | 302 (48.8) | 732 (73.6) | 0.528 | 278 (57.9) | 275 (57.1 | -0.013 |
| Uterine body invasion | 39 (6.3) | 126 (12.7) | 0.219 | 37 (7.7) | 41 (8.5) | -0.031 |
| Vaginal invasion | 71 (11.5) | 222 (22.3) | 0.293 | 70 (14.6) | 67 (14.0) | -0.018 |
| High-volume center | 215 (34.7) | 278 (28.0) | -0.146 | 142 (29.6) | 139 (29.0) | -0.0137 |

SCC, squamous cell carcinoma; LVSI, lymphovascular space invasion.

Table S2 Types of chemotherapy regimens

| Types of regimens | Number (%) |
| --- | --- |
| PTX/DTX + CBDCA/CDDP | 176 (51.8) |
| CPT-11 + CDGP | 58 (17.1) |
| BLM + VCR + MMC + CDDP | 20 (5.9) |
| MMC + VP-16 + CDDP ± Epi-ADR | 15 (4.4) |
| Others* | 67 (19.7) |
| No data | 4 (1.2) |

PTX, paclitaxel; DTX, docetaxel; CBDCA, carboplatin; CDDP, cisplatin; CPT-11, irinotecan; CDGP, nedaplatin; BLM, bleomycin; VCR, vincristine; MMC, mitomycin; VP-16, etoposide; Epi-ADR, 4’-Epi-Adriamycin; *, includes more than two regimens.

Table S3. Patients’ characteristics according to types of adjuvant therapies

|  | Before propensity score matching | | | After propensity score matching | | |
| --- | --- | --- | --- | --- | --- | --- |
|  | Radiation  n = 654 | Chemotherapy  n = 340 | Standardized differences | Radiation  n = 311 | Chemotherapy  n = 311 | Standardized differences |
| Age, mean  (Standard deviation) | 48.4 (12.3) | 47.2 (11.9) | -0.103 | 48.3 (11.8) | 47.2 (12.1) | -0.091 |
| Histology SCC | 488 (74.6) | 163 (47.9) | -0.569 | 164 (52.7) | 163 (52.4) | -0.006 |
| Size |  |  |  |  |  |  |
| 2 to <4 cm | 312 (47.7) | 180 (52.9) | 0.105 | 181 (58.2) | 166 (53.4) | -0.097 |
| ≥4 cm | 271 (41.4) | 105 (30.9) | -0.221 | 87 (28.0) | 97 (31.2) | 0.07 |
| LVSI positive | 493 (75.4) | 254 (74.7) | -0.016 | 239 (76.8) | 233 (74.9) | -0.045 |
| Cervical stromal invasion ≥1/2 | 498 (76.1) | 234 (68.8) | -0.165 | 223 (72.7) | 218 (70.1) | -0.035 |
| Uterine body invasion | 84 (12.8) | 42 (12.4) | -0.015 | 46 (14.8) | 39 (12.5) | -0.066 |
| Vaginal invasion | 172 (26.3) | 50 (14.7) | -0.290 | 57 (18.3) | 50 (16.1) | -0.060 |
| High-volume center | 204 (31.2) | 74 (21.8) | -0.215 | 70 (22.5) | 69 (22.2) | -0.008 |

SCC, squamous cell carcinoma; LVSI, lymphovascular space invasion.

Table S4A. Patients’ background characteristics according to types of adjuvant therapies in large tumor-sized group

|  | Before propensity score matching | | | After propensity score matching | | |
| --- | --- | --- | --- | --- | --- | --- |
|  | Radiation  n = 583 | Chemotherapy  n = 285 | Standardized differences | Radiation  n = 264 | Chemotherapy  n = 264 | Standardized differences |
| Age, mean  (Standard deviation) | 48.6 (12.1) | 47.0 (11.9) | -0.13 | 48.4 (12.2) | 47 (12.0) | -0.117 |
| Histology SCC | 436 (74.8) | 139 (48.8) | -0.56 | 140 (53.0) | 139 (52.6) | -0.008 |
| Size |  |  |  |  |  |  |
| 2 to <4 cm | 312 (53.5) | 180 (63.2) | 0.197 | 172 (65.5) | 166 (62.9) | -0.055 |
| ≥4 cm | 271 (46.5) | 105 (36.8) | -0.197 | 91 (34.5) | 98 (37.1) | 0.055 |
| LVSI positive | 430 (73.7) | 208 (73.0) | -0.018 | 196 (74.2) | 192 (72.7) | -0.034 |
| Cervical stromal invasion ≥1/2 | 460 (78.9) | 209 (73.3) | -0.131 | 196 (74.2) | 197 (74.6) | 0.009 |
| Uterine body invasion | 81 (13.9) | 37 (13.0) | -0.027 | 43 (16.3) | 35 (13.3) | -0.085 |
| Vaginal invasion | 159 (27.3) | 43 (15.1) | -0.302 | 43 (16.3) | 43 (16.3) | 0 |
| High-volume center | 186 (31.9) | 66 (23.2) | -0.197 | 66 (25) | 64 (24.2) | -0.018 |

SCC, squamous cell carcinoma; LVSI, lymphovascular space invasion.

Table S4B. Patients’ background characteristics according to types of adjuvant therapies in small tumor-sized group

|  | Before propensity score matching | | | After propensity score matching | | |
| --- | --- | --- | --- | --- | --- | --- |
|  | Radiation  n = 71 | Chemotherapy  n = 55 | Standardized differences | Radiation  n = 41 | Chemotherapy  n = 41 | Standardized differences |
| Age, mean  (Standard deviation) | 47.2 (13.6) | 48.3 (12.1) | 0.080 | 44.9 (12.6) | 48.2 (12.4) | 0.269 |
| Histology SCC | 52 (73.2) | 24 (43.6) | -0.630 | 24 (58.5) | 22 (53.7) | -0.098 |
| LVSI positive | 63 (88.7) | 46 (83.6) | -0.148 | 36 (87.8) | 35 (85.4) | -0.072 |
| Cervical stromal invasion ≥1/2 | 38 (53.5) | 25 (45.5) | -0.162 | 18 (43.9) | 20 (48.8) | 0.098 |
| Uterine body invasion | 3 (4.2) | 5 (9.1) | 0.196 | 3 (7.3) | 4 (9.8) | 0.087 |
| Vaginal invasion | 13 (18.3) | 7 (12.7) | -0.155 | 6 (14.6) | 6 (14.6) | 0 |
| High-volume center | 18 (25.4) | 8 (14.5) | -0.273 | 4 (9.8) | 5 (12.2) | 0.078 |

SCC, squamous cell carcinoma; LVSI, lymphovascular space invasion.
